# Laryngeal non-Hodgkin lymphoma presenting as throat foreign body sensation: A case report

**DOI:** 10.1097/MD.0000000000043132

**Published:** 2025-06-27

**Authors:** Hongming Liao, Ai He, Fengbo Yan, Benchao He

**Affiliations:** a Department of Otolaryngology Head and Neck Surgery, Tianmen First People’s Hospital, Tianmen, Hubei Province, China.

**Keywords:** immunohistochemistry, larynx, malignant lymphoma, Non-Hodgkin Lymphoma

## Abstract

**Rationale:**

Laryngeal lymphoma is an extremely rare malignancy, representing <1% of all laryngeal tumors. Diffuse large B-cell lymphoma (DLBCL) is the most common histologic subtype. Due to its rarity and nonspecific symptoms, early diagnosis and timely treatment are critical.

**Patient concerns:**

A 67-year-old male presented with progressive hoarseness and a persistent sensation of a foreign body in the throat that had developed over several weeks.

**Diagnoses:**

Laryngoscopy revealed a submucosal mass involving the glottis. Contrast-enhanced computed tomography demonstrated soft tissue infiltration. Surgical excision was performed, and histopathological evaluation confirmed the diagnosis of diffuse large B-cell lymphoma of the larynx.

**Interventions:**

The patient received systemic chemotherapy tailored to the staging and anatomical location of the lymphoma, following current DLBCL treatment guidelines.

**Outcomes:**

The patient experienced significant symptom relief after chemotherapy, with resolution of hoarseness and foreign body sensation. No recurrence was observed during the 2-month follow-up period.

**Lessons:**

This case highlights the importance of considering lymphoma in the differential diagnosis of persistent hoarseness and throat discomfort. Early biopsy and multidisciplinary intervention are essential to achieve favorable outcomes in patients with rare laryngeal DLBCL.

## 1. Introduction

Primary non-Hodgkin lymphoma (NHL) is a rare malignancy,^[1]^ most commonly found in the gastrointestinal tract, with laryngeal involvement accounting for <1% of all laryngeal tumors.^[2]^ Although squamous cell carcinoma remains the predominant type of laryngeal cancer, laryngeal lymphoma should be considered an important differential diagnosis, particularly when patients present with atypical symptoms such as hoarseness or a foreign body sensation in the throat; Laryngeal NHL typically manifests as a painless, slowly enlarging mass, often leading to confusion with other more common laryngeal conditions such as carcinoma or benign lesions.^[3]^ The majority of laryngeal lymphomas are of B-cell origin, with diffuse large B-cell lymphoma (DLBCL) being the most common subtype.^[4]^ Due to the limitations of imaging in distinguishing lymphoma from other neoplastic or inflammatory conditions, a definitive diagnosis requires histopathological examination, often through biopsy, followed by immunohistochemical staining to identify the lymphoma subtype^[5]^; Unlike laryngeal squamous cell carcinoma, which is primarily treated with surgery, NHL is generally managed with chemotherapy and/or radiation therapy.^[6]^ This nonsurgical approach is essential, as surgery has limited efficacy in treating lymphoma and may lead to unnecessary complications. Chemotherapy typically yields a favorable response, resulting in improved survival rates. This case report highlights a rare presentation of primary laryngeal NHL, emphasizing its clinical features, diagnostic challenges, and treatment outcomes. Early diagnosis and appropriate management are critical for improving patient prognosis in this uncommon malignancy.

## 2. Case presentation

### 2.1. History

A 67-year-old male patient presented to the Department of Otolaryngology on August 28, 2024, with complaints of persistent foreign body sensation in the throat and hoarseness. He had a significant history of long-term smoking, alcohol consumption, and irregular eating habits. These symptoms had progressively worsened over the past several weeks, prompting the patient to seek medical attention. This study was approved by the Ethics Committee of Tianmen First People’s Hospital (approval number: 20240336). The full name of the ethics committee is the Ethics Committee of Tianmen First People’s Hospital. In addition, the patient has provided written informed consent for the publication of this case.

### 2.2. Examination findings

**Figure 1.**
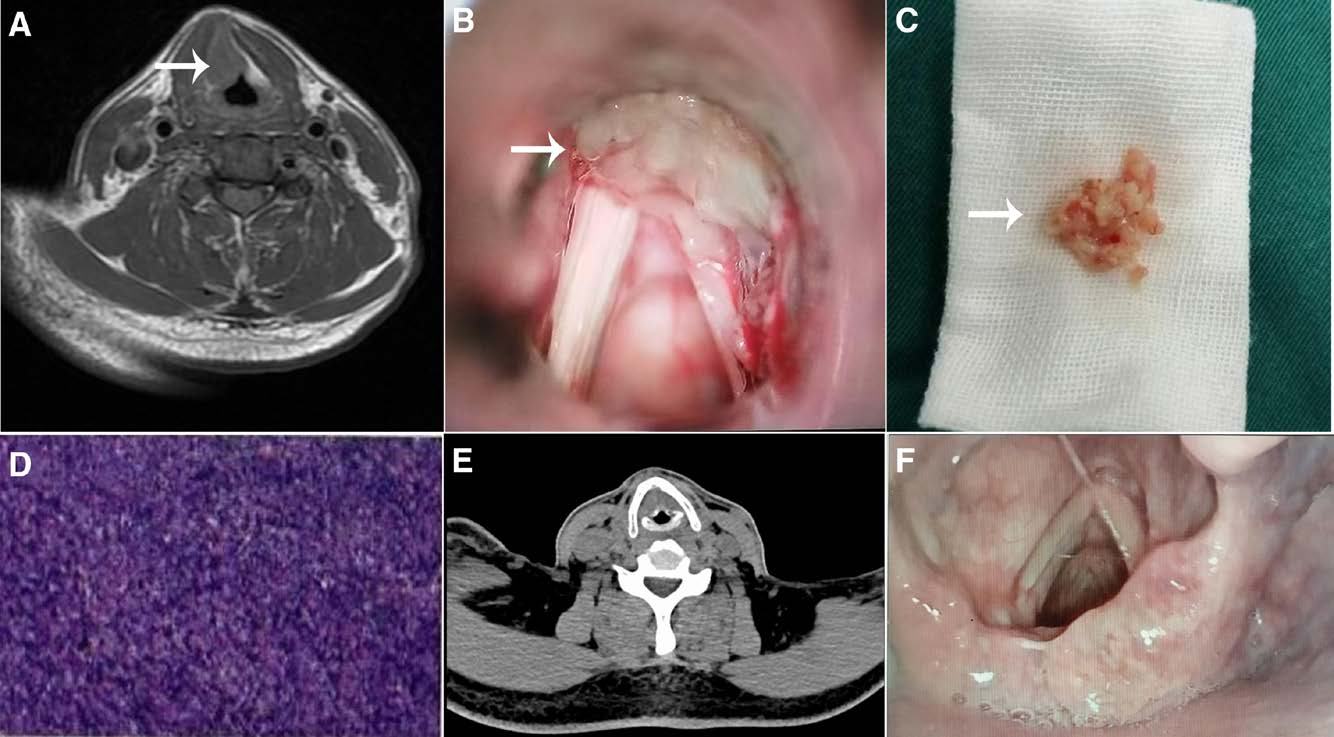
Treatment course of laryngeal non-Hodgkin lymphoma. (A) The preoperative neck MRI results, with the mass indicated by the arrow. (B) The intraoperative findings showed the mass, indicated by the arrow. (C) Intraoperative specimen of the resected mass. (D) Postoperative pathological results. (E) Postoperative follow-up neck CT showed no mass in the thyroid area. (F) Two months postoperatively, follow-up flexible laryngoscopy showed no recurrence.

On outpatient examination, electronic laryngoscopy revealed a mass on the right ventricular fold, partially covering the anterior commissure. The appearance of the mass suggested a benign lesion. However, further diagnostic workup, including enhanced magnetic resonance imaging of the larynx, showed a nodular lesion on the right vocal fold extending to the anterior commissure, raising suspicion for malignancy. Abnormal signals were also noted in the upper mediastinum, which were suggestive of a cystic lesion (Fig. 1A).

### 2.3. Treatment sources

On August 31, 2024, the patient underwent surgical resection of the right ventricular fold tumor under general anesthesia. Intraoperative findings confirmed that the tumor originated from the right ventricular fold, with partial involvement of the anterior commissure. The excision was performed using high-frequency electrosurgery, and a grayish-white mass was observed. A portion of the tumor was sent for histopathological examination (Fig. 1B and C). Postoperative pathology confirmed a diagnosis of non-Hodgkin B-cell lymphoma (aggressive type), with immunohistochemistry confirming diffuse large B-cell lymphoma (non-germinal center phenotype) (Fig. 1D).

### 2.4. Follow-up

Following surgery, postoperative 18F-fluorodeoxyglucose positron emission tomography on September 11, 2024, showed no abnormal findings in the pharyngeal region, with no evidence of other tumors. Small lymph nodes in the cervical, mediastinal, and axillary regions showed low glucose metabolism, consistent with inflammation or reactive lymphadenopathy. A follow-up neck computed tomography (CT) scan revealed glottic swelling and a narrowed glottic space (Fig. 1E), while gastrointestinal endoscopy did not show any distant metastasis. The patient was referred to hematology for bone marrow aspiration, which revealed erythroid hyperplasia but no other abnormalities. Due to financial constraints, he opted for treatment with rituximab and lenalidomide chemotherapy. Follow-up electronic laryngoscopy 2 months post-surgery showed no recurrence (Fig. 1F), and the patient remains under ongoing follow-up care.

## 3. Discussion

In 1934, MacKenty reported the first case of primary NHL of the larynx.^[2,7]^ Laryngeal non-Hodgkin lymphoma, particularly DLBCL of the larynx, is a rare form that primarily affects middle-aged and elderly individuals, with a higher incidence in males than females.^[8]^ The literature reports that the incidence rate of laryngeal NHL is relatively low, and its clinical manifestations are usually lack of specificity, leading to diagnostic challenges.^[9,10]^ Most cases are found in adults, with a predilection for male patients and typically older individuals. The involvement of the larynx is often associated with extranodal lymphoma, meaning the lymphoma arises outside the lymph nodes, frequently affecting the Waldeyer ring, tonsils, and nasopharynx.

While NHL can affect various regions of the head and neck, laryngeal involvement is exceedingly rare, with reported incidence rates ranging from approximately 1% to 17% of all head and neck lymphomas.^[11,12]^ Common symptoms include dyspnea, dysphagia, voice changes, hoarseness, and cervical lymphadenopathy. The exact cause of laryngeal DLBCL remains unclear, though it is associated with factors such as Epstein-Barr virus infection, methotrexate therapy in rheumatoid arthritis patients, HIV infection, and chronic hepatitis B.^[13]^ Treatment typically involves chemotherapy, with the Rituximab, Cyclophosphamide, Doxorubicin, Vincristine, and Prednisone regimen (rituximab combined with chemotherapy) being the most common approach, and radiation therapy often used as an adjunct to enhance outcomes.^[14]^ Early detection and timely treatment generally improve prognosis, but the tumor’s subtle location can delay diagnosis and impact outcomes; thus, persistent laryngeal symptoms in middle-aged and elderly patients warrant careful evaluation.

This case highlights the rare presentation of laryngeal NHL, initially manifested as a foreign body sensation in the throat. While such symptoms are typically associated with benign conditions like pharyngolaryngeal reflux or upper respiratory infections, the patient did not show signs of inflammation, infection, or common benign laryngeal lesions. Endoscopic examination revealed a laryngeal mass, initially suspected to be benign, but biopsy and pathology confirmed non-Hodgkin lymphoma. The discrepancy between the imaging and pathological findings may be related to the clinical presentation and the diverse imaging characteristics of non-Hodgkin lymphoma. Laryngeal lymphoma often presents subtly, with symptoms such as hoarseness, dysphagia, and dyspnea, which overlap with more common laryngeal diseases. As seen in this case, these nonspecific symptoms are also common in benign conditions like pharyngolaryngeal reflux. Moreover, laryngeal lymphoma may present as an isolated mass without systemic symptoms like weight loss or fever, typically seen in more aggressive lymphomas. The absence of distinctive features, such as rapid tumor growth or severe pain, often leads to delayed diagnosis. The patient in this case underwent surgical excision followed by chemotherapy, resulting in significant improvement and no recurrence at a 2-month follow-up. This case underscores the importance of considering rare malignancies in the differential diagnosis of laryngeal masses, particularly when symptoms do not align with common benign conditions. Early detection and appropriate treatment are crucial for improving prognosis, offering valuable insights for clinical education and research on laryngeal non-Hodgkin lymphoma.

Diagnosing NHL can be challenging due to its similarity to more common conditions. As noted by Hu et al (2024),^[10]^ laryngeal lymphoma often presents with clinical features resembling chronic laryngeal inflammation, which further complicates the diagnostic process. Laryngeal NHL is predominantly found in the supraglottic region, likely due to the rich lymphoid tissue in this area, followed by the glottis and subglottic regions. Endoscopically, NHL typically presents as a smooth, non-ulcerated mass. Imaging techniques such as CT and magnetic resonance imaging may identify a well-defined mass, but these findings are not always indicative of malignancy, especially in the absence of lymphadenopathy. Nonetheless, these imaging modalities are valuable for assessing tumor infiltration. The role of positron emission tomography–CT in diagnosing laryngeal NHL remains debated.^[15–17]^ As illustrated in this case, biopsy and pathological examination are essential for confirming the diagnosis and guiding therapeutic decisions.

This case contributes significantly to the understanding of NHL by emphasizing the importance of considering rare malignancies in patients with unexplained laryngeal symptoms. While a foreign body sensation is not pathognomonic for lymphoma, its presentation as the primary symptom in this case is relatively unusual and highlights a diagnostic challenge. Typically, such symptoms are more commonly associated with benign conditions like pharyngolaryngeal reflux or upper respiratory infections. However, this case underscores the necessity for clinicians to maintain a high index of suspicion for malignancies, even when clinical symptoms are nonspecific. It is crucial to recognize that although the laryngeal NHL may not initially present with the typical signs of malignancy—such as rapid tumor growth, significant pain, or lymphadenopathy—it can still lead to serious health implications if not promptly diagnosed. This case further highlights the importance of a comprehensive diagnostic workup, which included flexible laryngoscopy, biopsy, and histopathological examination. Such a systematic approach allowed for the identification of a rare and easily overlooked condition. Indeed, this case serves as a reminder that a thorough and meticulous evaluation is key to identifying atypical presentations that could otherwise be missed. A timely diagnosis of laryngeal NHL is critical, as its management and prognosis differ significantly from those of benign laryngeal conditions or more common malignancies like squamous cell carcinoma. Early recognition and appropriate treatment can significantly improve patient outcomes and reduce the risk of complications, making this case a valuable contribution to both clinical education and research in the field of head and neck oncology.

One limitation of this case study is that it involves a single patient, which limits the generalizability of the findings. Future research should focus on larger cohort studies to better understand the clinical features, management strategies, and outcomes of laryngeal NHL. In particular, more studies are needed to investigate the optimal diagnostic protocols for laryngeal lymphoma, including the roles of imaging, biopsy techniques, and molecular markers.

Additionally, the treatment of laryngeal NHL is still a subject of ongoing debate. Chemotherapy, radiation therapy, and surgical management have all been used with varying success, but there is no consensus on the best approach. Future studies should focus on identifying prognostic factors that can guide treatment decisions, including the role of immunohistochemistry, genetic profiling, and response to initial therapies.

Lastly, given the rarity of laryngeal NHL, it is crucial for clinicians to collaborate and share case reports to improve awareness and understanding of this condition. The findings from this case can help establish more comprehensive diagnostic criteria and treatment algorithms, particularly for clinicians in regions with limited access to advanced diagnostic tools or specialized cancer care.

## 4. Conclusions

Laryngeal NHL, though rare, should be considered in the differential diagnosis when a patient presents with symptoms such as foreign body sensation, hoarseness, or dysphagia, especially when these symptoms do not resolve with typical conservative management. This case highlights the importance of thorough clinical evaluation, including biopsy and histopathology, to establish an accurate diagnosis. While the management of laryngeal NHL is still evolving, early recognition and intervention are key to improving patient outcomes. Future research should aim to refine diagnostic strategies, treatment protocols, and prognostic factors, ultimately improving the care of patients with this uncommon condition.

## Author contributions

**Data curation:** Ai He.

**Formal analysis:** Benchao He.

**Writing – original draft:** Hongming Liao.

**Writing – review & editing:** Fengbo Yan.
